# Small molecule WDR5 inhibitors down-regulate lncRNA expression†

**DOI:** 10.1039/d3md00605k

**Published:** 2024-01-10

**Authors:** Jen-Yao Chang, Cora Neugebauer, Anne Mues genannt Koers, Peter 't Hart

**Affiliations:** a Chemical Genomics Centre of the Max Planck Society, Max Planck Institute of Molecular Physiology Otto-Hahn-Strasse 11 44227 Dortmund Germany peter.t-hart@mpi-dortmund.mpg.de

## Abstract

WD repeat domain 5 (WDR5) plays an important role as a scaffold protein in both protein–protein and RNA–protein complexes involved in epigenetic gene regulation. In particular, some of these lncRNAs were reported to regulate the expression of genes in *cis* as well as themselves through binding WDR5. In this report, we investigate the two known binding sites of WDR5 in relation to lncRNA binding and expression. The WBM binding site mediates both protein–protein and lncRNA–protein interactions while the WIN site, which is on the opposite side of the protein, is only known to mediate protein–protein interactions. To dissect the function of different binding sites on WDR5, we characterized them with selective peptide ligands using fluorescence polarization and used these to demonstrate the selectivity of small molecule inhibitors of these two major binding sites. RNA immunoprecipitation experiments were performed to show that lncRNA–WDR5 complex formation could be interrupted using a WBM site inhibitor. Finally, we demonstrated that WDR5 regulated lncRNAs are down regulated with different sensitivity toward the corresponding inhibitors, demonstrating the potential of targeting lncRNA–protein interactions to reduce oncogenic lncRNA expression.

## Abstract 2

WDR5 inhibitors selective for either one of its binding sites shed light on its role in regulation of lncRNA expression.

## Graphical abstract

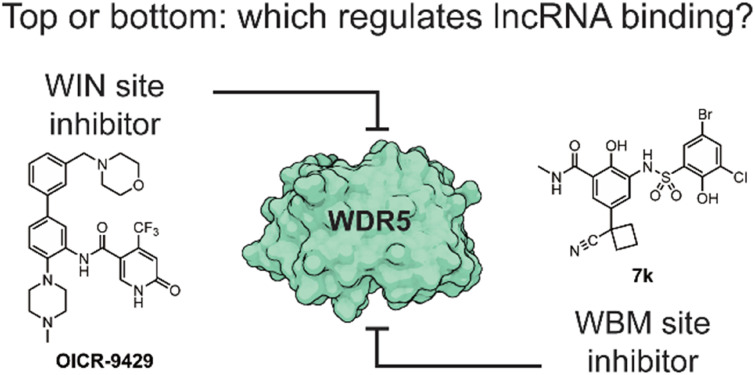

## Introduction

**Fig. 1:**
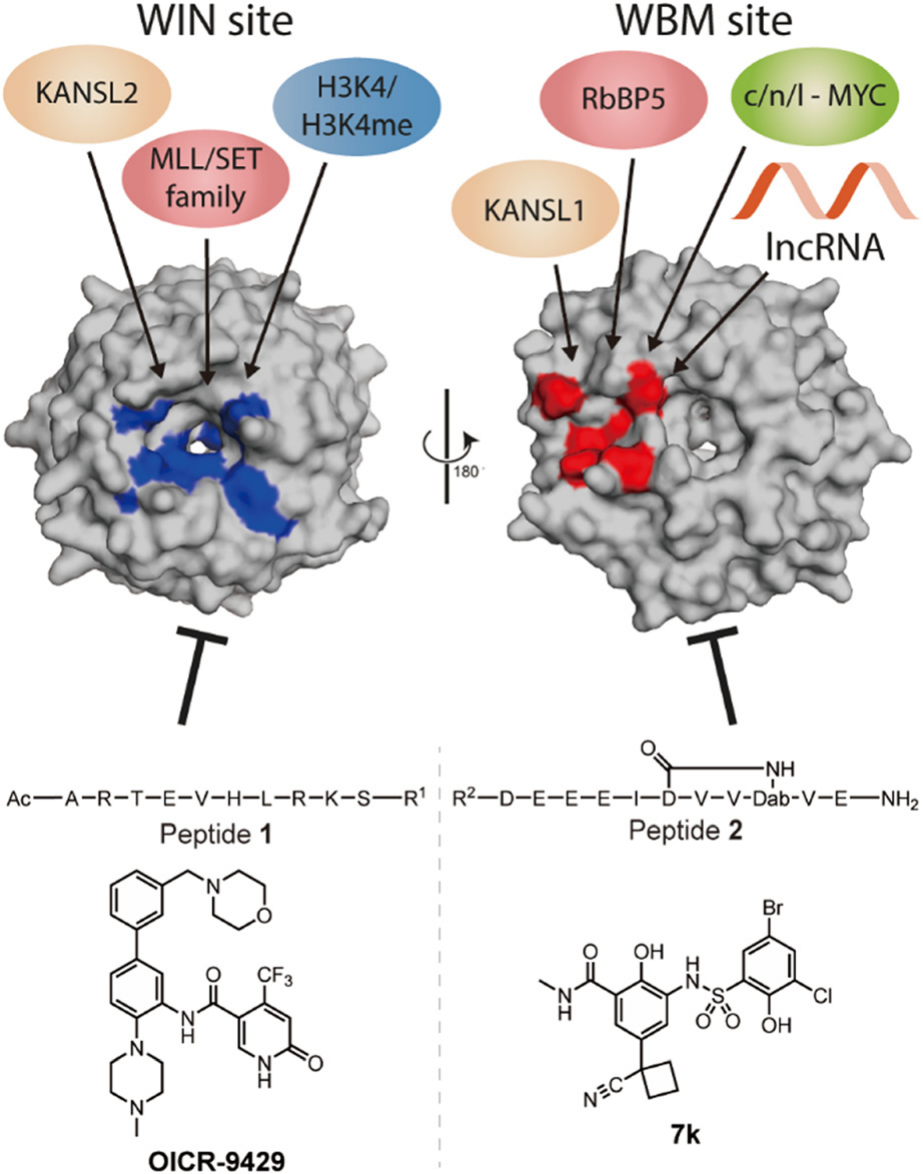
Crystal structure (PDB: 8Q1N) of WDR5 and the reported binding partners for the corresponding binding sites. A65, S91, D107, F133, Y191, Y260, F263 are coloured in blue to indicate the location of WIN binding pocket; N225, Y228, L240, F266, V268, Q289 are coloured in red to indicate the location of WBM binding pocket.

WD repeat domain 5 (WDR5) is a protein consisting of seven repeating units of the WD domain, forming an iconic donut shaped protein structure (Fig. 1).^1^ It usually acts as a scaffold protein for larger protein complex formation, including various epigenetic modulating complexes such as the MLL-COMPASS complex, NSL complex, NuRD complex, and the MYC-MAX complex.^2–6^ In addition to being a scaffold in protein–protein complexes, it can also form long noncoding RNA (lncRNA)–protein complexes to modulate histone modifications, leading to changes in gene expression.^7,8^ For example, *HOTTIP* is a lncRNA that requires WDR5 to activate the late HOXA gene cluster as well as its own expression to maintain cell self-renewal and pluripotency.^7,8^ To do so, *HOTTIP* acts as a guide that recruits WDR5 to chromatin, followed by recruitment of the MLL complex components to initiate histone 3 lysine 4 methylation at the target genes.^8,9^ Dysregulation of *HOTTIP* is correlated to cancer progression and could become an interesting therapeutic target.^8–11^ Although small-interfering RNA (siRNA) can selectively silence their target lncRNAs, they are limited in their ability to distribute and permeate cells.^12^ An alternative strategy to influence oncogenic lncRNA levels such as *HOTTIP* is not through direct targeting, but rather by targeting the proteins that are part of their mode of action. This was highlighted in the report by Wang *et al.*, who demonstrated that knock down of WDR5 lead to reduced expression levels of *HOTTIP*.^7^ The same group later reported how *HOTTIP* recognizes the WBM site on WDR5.^8^ Their results suggested that WDR5 is required for *HOTTIP* expression and that targeting the *HOTTIP*–WDR5 interaction with competitive inhibitors could be an interesting therapeutic strategy. Besides *HOTTIP*, a report by Subhash *et al.* demonstrated WDR5 binds many more lncRNAs including ones that have reported oncogenic activity further highlighting the potential of inhibiting such interactions.^13^ Unlike protein–protein interactions (PPI), RNA–protein interactions (RPI) often lack structural information to assist in the design process of inhibitors. In addition, WDR5 has two major binding pockets, the WIN site, and the WBM site, making the situation more complicated for designing effective and selective inhibitors. Several PPI inhibitors have been developed and studied for their ability to target protein–WDR5 interactions, *e.g.* Karatas *et al.* reported an optimized sequence that has sub-nanomolar binding affinity to the WIN site, while Grebien *et al.* demonstrated that **OICR-9429**, a small molecule inhibitor for the WIN pocket, can antagonize the WDR5-MLL interaction *in cellulo*.^14,15^ On the other hand, the WBM site is much less explored but is promising for its role in the MYC dependent pathway. Macdonald *et al.* demonstrated that their optimized WBM inhibitor 7k could reduce cMYC enrichment in WDR5 pull-down experiments,^16^ while Ding *et al.*, reported that their WDR5-cMYC interaction inhibitor leads to growth inhibition in several cancer cells.^17,18^ Recently, we reported that targeting the WBM site with a macrocyclic peptide could reduce lncRNA enrichment by WDR5 pull-down *in vitro.*^19^ Here, we aim to bring these details together to provide a clear picture of the possibility of targeting lncRNA–WDR5 interactions as a therapeutic strategy by using well described inhibitors of the two binding sites on WDR5.

## Results

**Fig. 2:**
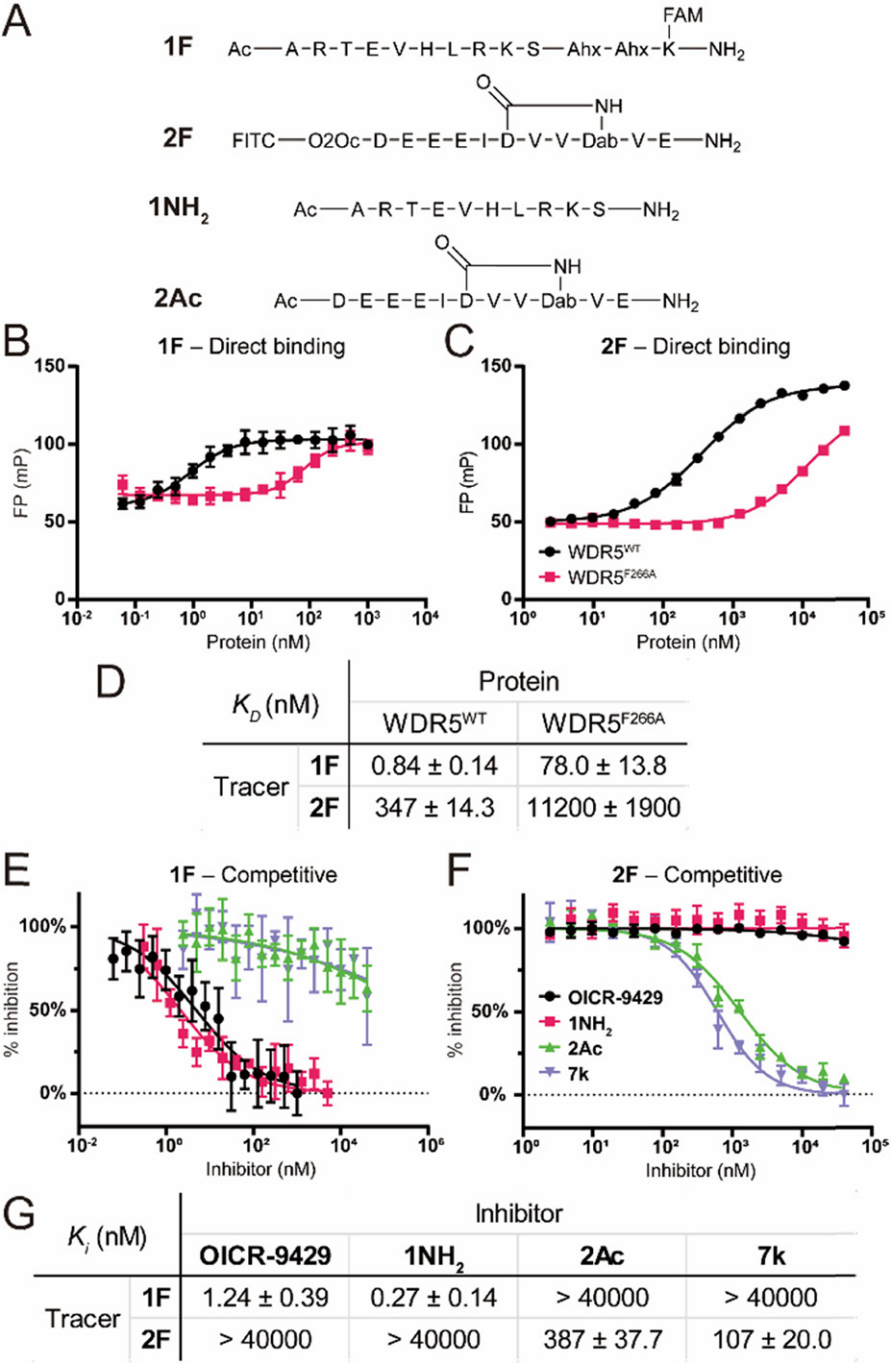
Fluorescence polarization assay using peptides 1F and 2F as tracers. A: Chemical structure of the used peptides. B: Titration of WDR5^WT/F266A^ against 1F. C: Titration of WDR5^WT/F266A^ against 2F. D: Binding affinities of peptides 1F and 2F. E: Competitive fluorescence polarization experiments at the WIN site. Inhibitors were titrated against a fixed concentration of 1F–WDR5^WT^ complex. F: Competitive experiment at the WBM site. Inhibitors were titrated against a fixed concentration of 2F–WDR5^WT^ complex. G: *K*_I_ values measured for all compounds determined in the competitive fluorescence polarization experiments. All FP experiments were performed as two biological replicates of two technical replicates each. *K*_I_ values are calculated as average of all four replicates and errors are reported as the standard deviation.

To characterize the binding affinity of the two major binding sites on wildtype FLAG-WDR5 (WDR5^WT^), a direct fluorescence polarization (FP) experiment was performed with two selective peptide binding probes, peptide 1F for the WIN site, and peptide 2F for the WBM site (Fig. 2A).^14^ The observed affinity for both binding sites was similar to the previously reported values (Fig. 2B–D). The commonly used mutant WDR5^F266A^ was evaluated using the same protocol to reveal its effect on the two binding sites. This mutant was reported to have a lower lncRNA binding affinity, while retaining the ability for MLL-complex formation and the corresponding histone methylation potential (Fig. 2B–D).^8^ Since lncRNA binding takes place *via* the WBM site the reduced affinity of peptide 2 was expected. However, WDR5^F266A^ also shows a 93-fold lower affinity for the WIN-site binding peptide when compared to WDR5^WT^, meaning that the mutation can also retard protein complex formation although the affinity is still in the nanomolar range (Fig. 2B and D). To rule out effects of the fluorescent labels we evaluated them using the unlabelled peptides **1NH**_**2**_ and **2Ac** as competitors (Fig. 2A). The results confirmed that the tracers recognized their target sites in a specific manner (Fig. 2E–G) as the obtained *K*_I_ values of the unlabelled peptides were similar to the *K*_D_ values of the labelled peptides. Furthermore, the acetylated WIN-site peptide **1NH**_**2**_ does not inhibit the WBM-site and *vice versa* meaning that these two tracers were orthogonal.

Besides the site selective peptides, we also evaluated two small molecules, **OICR-9429** and 7k, which were designed to be selective for the WIN and WBM site respectively, but their selectivity toward the other binding site was not evaluated (Fig. 2E–G).^15,16^ Indeed, we did not observe any crossover inhibition for the small molecules at the opposite binding sites (Fig. 2G).

**Fig. 3:**
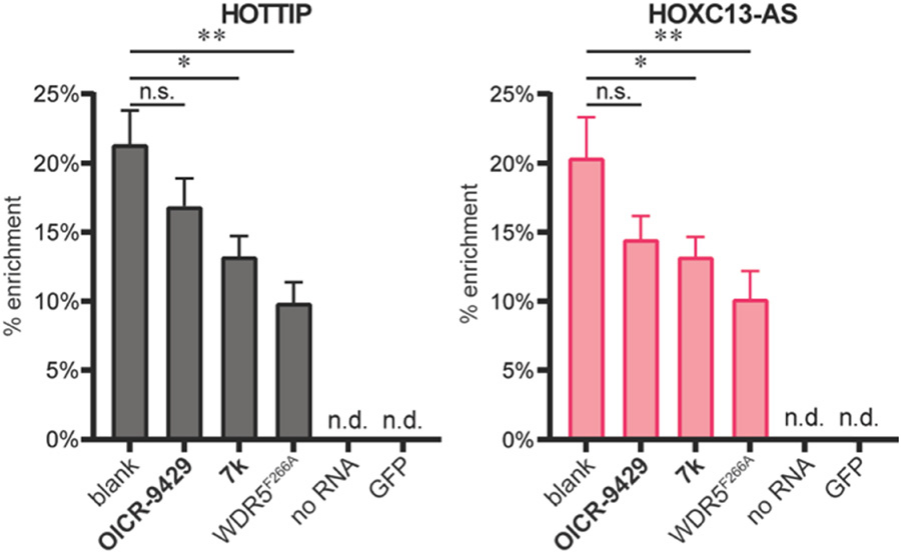
*iv*-RIP results using RNA isolates from U-2 OS cells and WDR5^WT^, WDR^F266A^, or GFP. Compounds **OICR-9429** and 7k were tested at 10 μM in combination with WDR5^WT^. The no RNA control was tested with WDR5^WT^. n.s.: *p* > 0.05, *: 0.05 ≥ *p* > 0.01, **: 0.01 ≥ *p* > 0.001.

After characterizing the binding site selectivity of **OICR-9429** and 7k, we evaluated their ability to compete with lncRNA–WDR5 complex formation. In a report from our group, we demonstrated that peptides were able to inhibit lncRNA–protein interactions *in vitro*, and only inhibitors for targeting the WBM site can reduce RNA–WDR5 complex formation.^19^ Here the same strategy was applied using the small molecules, utilizing competitive *in vitro* RNA immunoprecipitation (*iv*-RIP) to verify the influence of small molecules on lncRNA–protein complex formation. To this end, cellular RNA extracts were incubated with either DMSO or compound, in the presence of FLAG-WDR5^WT^ or FLAG-WDR5^F266A^, and immunoprecipitated using an anti-FLAG antibody. The coprecipitated RNA was then isolated and analysed using RT-qPCR. In this experiment, two lncRNAs, *HOTTIP* and *HOXC13-AS*, were monitored for their enrichment from RNA extracts from U-2 OS cells because they were both reported to be sensitive to WDR5 knockdown.^7,13^ The results demonstrated that molecule 7k, the direct inhibitor for the WBM site, could inhibit *HOTTIP*–WDR5^WT^ and *HOXC13-AS*–WDR5^WT^ complex formation (Fig. 3) but the effect was moderate. Although we did not observe inhibition of the WBM site from **OICR-9429** in the FP experiments, it was able to reduce RNA binding albeit not significantly. A no-RNA control as well as a FLAG-GFP control were included to demonstrate that RNA enrichment was dependent on the presence of a WDR5 variant. WDR5^F266A^ also showed a reduced capability of enrichment for RNA, even though WDR5^F266A^ itself was more efficiently enriched than WDR5^WT^ (Fig. S6†).

**Fig. 4:**
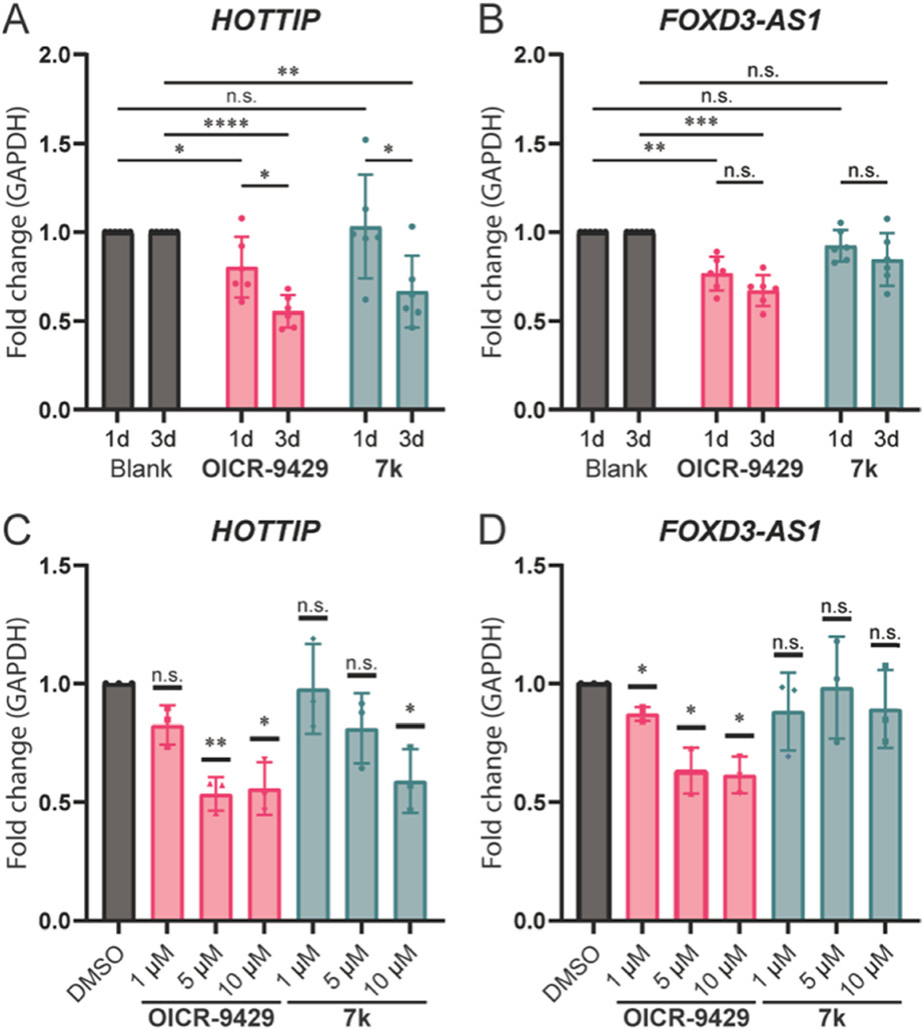
lncRNA expression levels under various treatment conditions. RT-qPCR results were analysed using the 2^−ΔΔCt^ method.^20^ A: Expression levels of *HOTTIP* after one/three-days incubation, Blank was treated with 0.1% DMSO while compounds were used at 10 μM. B: Expression levels of *FOXD3-AS1* after one/three-days incubation time, Blank was treated with 0.1% DMSO while compounds were used at 10 μM. C: Expression level of *HOTTIP* after treatment with 1/5/10 μM compound of interest for three days. D: Expression level of *FOXD3-AS1* after treatment with 1/5/10 μM compound of interest for three days. n.s.: *p* > 0.05, *: 0.05 ≥ *p* > 0.01, **: 0.01 ≥ *p* > 0.001, ***: 0.001 ≥ *p* > 0.0001, ****: 0.0001 ≥ *p*.

Wang *et al.* reported that knocking down WDR5 could lead to the downregulation of *HOTTIP* and several genes around the late HOXA region.^7^ We hypothesized that we could use the small molecule inhibitors to verify if a direct lncRNA–WDR5 interaction is required for cells to maintain lncRNA expression levels. **OICR-9429** and molecule 7k were used to antagonize the lncRNA–WDR5 complex in MDA-MB-231 cells, a triple-negative breast cancer cell line, and the expression level of *HOTTIP* was studied. Treatment with molecule 7k lead to down-regulation of *HOTTIP* after three days of treatment (Fig. 4A), showing that interrupting the *HOTTIP*–WDR5 interaction *in cellulo* indeed could lead to down-regulation of *HOTTIP* itself. After a one-day treatment no effect could be observed, indicating that the downregulation takes time to develop. Although **OICR-9429** did not inhibit lncRNA–WDR5 complex formation as strongly as molecule 7k, it is still able to reduce *HOTTIP* expression after three days of treatment.^21^ In addition, one-day treatment of **OICR-9429** has a small but significant effect. Taken together the data suggests that targeting WDR5 irrespective of the binding site leads to down-regulation of *HOTTIP*.

Besides *HOTTIP*, a second lncRNA, *FOXD3-AS1*, was analysed under the same conditions as it was previously described to be bound by WDR5,^13^ while *HOXC13-AS* was not detectable in MDA-MB-231 (Table S3†). It is worth noting that *FOXD3-AS1* does not show a time dependency after prolonging the treatment from one to three days (Fig. 4B). Treatment with **OICR-9429** lead to a 20% reduction of expression of *FOXD3-AS1*, while treatment with molecule 7k did not provide significant changes, indicating that *FOXD3-AS1* was not sensitive to the disruption of *FOXD3-AS1*–WDR5 complex, but still depend on a fully functional WDR5 with a free WIN binding pocket.

In addition to time dependency, concentration dependency was also tested. The treatment time was set to three days to ensure a clearly observable effect. The effect of treatment with **OICR-9429** reached a maximum at 5 μM for both *HOTTIP* and *FOXD3-AS1*, while 1 μM of **OICR-9429** was not enough to reduce the expression of *HOTTIP* significantly (Fig. 4C and D). On the other hand, a concentration dependent effect for molecule 7k could only be observed for *HOTTIP*, and 10 μM was required to provide significant changes. Meanwhile, *FOXD3-AS1* is not sensitive to the treatment of molecule 7k in the concentration range used, similar to what we found in the time-dependent experiment.

## Conclusions

In this report, we investigated the roles of the two known binding sites (WIN and WBM) of WDR5 by competitive FP assays. The WIN and WBM sites did not show any allosteric influence on each other when the peptide-based tracers were used that were derived from native WDR5 protein binding partners. Furthermore, we tested the mutant WDR5^F266A^ to quantify the binding affinity and revealed that this mutation leads to a reduced affinity for its peptide binding partners similar as was reported for lncRNA. Although this was expected for the WBM site binding peptide, it is unclear why this mutation also affects binding of the WIN site targeting peptide 1F.

As determined by *iv*-RIP, molecule 7k could inhibit lncRNA–WDR5 formation to a level almost similar to the WDR5^F266A^ mutant. Based on previously described data, we hypothesized that interruption of the *HOTTIP*–WDR5 interaction could downregulate *HOTTIP*.^8^ Indeed, 7k was able to reduce *HOTTIP* expression but longer incubation times of three days were required to observe the effect. Interestingly, the same treatment did not influence *FOXD3-AS1* expression although a previous study published by Subhash *et al.* demonstrated that silencing WDR5 could.^13^ These results suggest that *FOXD3-AS1* may require the presence of WDR5 to maintain its expression, but not *FOXD3-AS1*–WDR5 complex formation.

In contrast to 7k, the WIN site inhibitor **OICR-9429** was not able to reduce lncRNA binding significantly in the *iv*-RIP experiments. Albert *et al.* proposed a potential allosteric control between the WIN and WBM sites through the interaction on one of the WD40 blades,^22^ which indicates a possible explanation of how WIN sites could interfere with the binding at WBM sites. However, more extensive structural and biochemical experiments will be required to support this hypothesis. Nevertheless, the ability of **OICR-9429** to antagonize the WDR5 supported histone methylation is likely the main reason for observing an overall downregulation of *HOTTIP* as well as *FOXD3-AS1*. A difference in time dependence was observed for the two lncRNAs (Fig. 4B) where the effect for *FOXD3-AS1* showed no difference between a one-day or three-day treatment, while the effect for *HOTTIP* increased after prolonged treatment.

By combining the fact that **OICR-9429** could downregulate *FOXD3-AS1* while 7k could not, but that 7k was able to downregulate *HOTTIP* it seems possible to selectively downregulate specific lncRNAs based on the targeted binding site. Considering the role *HOTTIP* plays in cancer development and the oncogenes that it controls, it is very interesting to see that it could be downregulated without a direct RNA silencing strategy, providing new possibilities in targeting selected oncogenic lncRNAs. These strategies can involve small molecules targeting epigenetic scaffolding proteins rather than RNA silencing approaches which typically suffer from difficulties with delivery.

## Author contributions

Jen-Yao Chang: conceptualization, methodology, validation, formal analysis, investigation, writing – original draft, visualization, supervision, funding acquisition Cora Neugebauer: methodology, validation, investigation Anne Mues: methodology, investigation Peter 't Hart: conceptualization, resources, writing – review & editing, visualization, supervision, project administration, funding acquisition.

## Conflicts of interest

There are no conflicts to declare.

## Supplementary Material

MD-015-D3MD00605K-s001
