# Supplementary material for: Small molecule WDR5 inhibitors down-regulate lncRNA expression
Source: RSC Med Chem. 2024 Jan 10;15(2):636–40. doi: 10.1039/d3md00605k (PMC10880924; doi:10.1039/d3md00605k)
Supplement: MD-015-D3MD00605K-s001 [file MD-015-D3MD00605K-s001.pdf]

Electronic Supplementary Material (ESI) for RSC Medicinal Chemistry.
This journal is © The Royal Society of Chemistry 2024

Supplemental information for

Small molecule WDR5 inhibitors down-regulate lncRNA expression

*Jen-Yao Chang, Cora Neugebauer, Anne Mues genannt Koers, Peter 't Hart**

Contents

Supplemental information for 1

Macrocyclic peptides as inhibitors of WDR5 -lncRNA interactions 1

General Information 4

Preparation of Small Molecules 5

1-(3-Bromo-4methoxyphenyl)cyclobutane-1-carbonitrile (S2) 5

Phenyl 5-(1-Cyanocyclobutyl)-2-methoxybenzoate (S3) 6

Phenyl 5-(1-Cyanocyclobutyl)-2-hydroxybenzoate (S4) 7

Phenyl 5-(1-Cyanocyclobutyl)-2-hydroxy-3-nitrobenzoate (S5) 8

Phenyl 3-Amino-5-(1-cyanocyclobutyl)-2-hydroxybenzoate (S6) 8

5-Bromo-3-chloro-2-hydroxybenzenesulfonyl Chloride (S8) 9

3-((5-Bromo-3-chloro-2-hydroxyphenyl)sulfonamido)-5-(1cyanocyclobutyl)-2-hydroxy-N-methylbenzamide (7k) 10

Preparation of peptides 11

Synthesis of WIN peptide 11

Synthesis of FAM-labeled WIN peptide (1F) 11

Synthesis of none-labeled WIN peptide ($1NH_2$) 12

Synthesis cMYC-cyclic peptide 12

Synthesis of FITC-labeled cMYC-cyclic peptide (2F) 13

Synthesis of none-labeled cMYC-cyclic peptide (2Ac) 13

Recombinant protein expression and purification 14

Fluorescence Polarization 14

FP measurement for direct binding assay 15

FP measurement for competitive assay 15

Cancer cell line 16

Total RNA isolation 16

RNA immunoprecipitation (RNA-IP) 16

lncRNA expression level assay 17

Quantitative reverse transcription-polymerase chain reaction (RT-qPCR) 17

Data analysis 17

Percentage of enrichment for RNA-IP 17
Fold change of gene expression level 18
Appendix 19
Buffer recipe list: 19
Primer List 19
HPLC analysis of compounds 20
Normal lncRNA expression level in different cell lines 22
Results of RIP 23
Results of cell treatment 24
Reference 27

General Information

All commercially available reagents and solvents were purchased and used directly without any further treatment unless specified. Solvents for chromatography were laboratory grade. Thin-layer chromatography (TLC) was performed using Supelco silica gel aluminum sheets with F_{254} indicator, visualized by irradiation with UV light. Column chromatography was performed using Acros Organics silica gel 60 Å (particle size 0.035 – 0.070 mm). Thermo Scientific HAAKE EK90 with an acetone bath in Dewar vessel was applied together to cool reactions to – 78 ^{o}C. Preparative HPLC was performed using BÜCHI Pure C-850 FlashPrep equipped with a C18 column (Macherey-Nagel, 5 µm, 125 x 21 mm, flow rate: 20 mL/min). Analytical HPLC-MS was performed by using Agilent 1200 Infinity II LC system equipped with a C18 column (Agilent Poroshell 120, 2.7 µm, 3 x 100 mm, flow rate: 0.4 mL/min, temperature: 33 ^{o}C) and Agilent InfinityLab LC/MSD G6125C, and the compounds were eluted with a linear gradient from 5% to 95% (percentage of B. A: 0.1% TFA in H2O; B: 0.1% TFA in ACN) over 20 min. The whole UV-VIS spectrum was recorded during the run, and the selected channel (210 / 254 nm), was used to plot and calculate the purity of the samples. High-resolution mass spectra were recorded on a LTQ-XL Orbitrap mass spectrometer coupled to an Accela HPLC System (HPLC column: Hypersyl GOLD, 50 mm x 1 mm, 1.9 µm). ^{1}H-NMR and ^{13}C-NMR, COSY-NMR, HSQC-NMR, HMBC-NMR were recorded on a Bruker Avance III HD NanoBay (400 MHz) or a Bruker Avance III HD (500 MHz, 600 MHz, 700 MHz) spectrometer in $CDCl_3$ or DMSO-d_6. Chemical shifts (d) are reported in ppm, coupling constants (J) are reported in Hertz (Hz), splitting patterns are indicated as s (singlet), d (doublet), t (triplet), q (quartet), m (multiplet). Gels were imaged using a Bio-Rad ChemiDoc MP imaging system. Reverse transcription (RT) was performed by Eppendorf Mastercycler ep gradient, and quantitative polymerase chain reaction (qPCR) was performed by Bio-Rad CFX Connect Real-Time PCR Detection System.

Preparation of Small Molecules

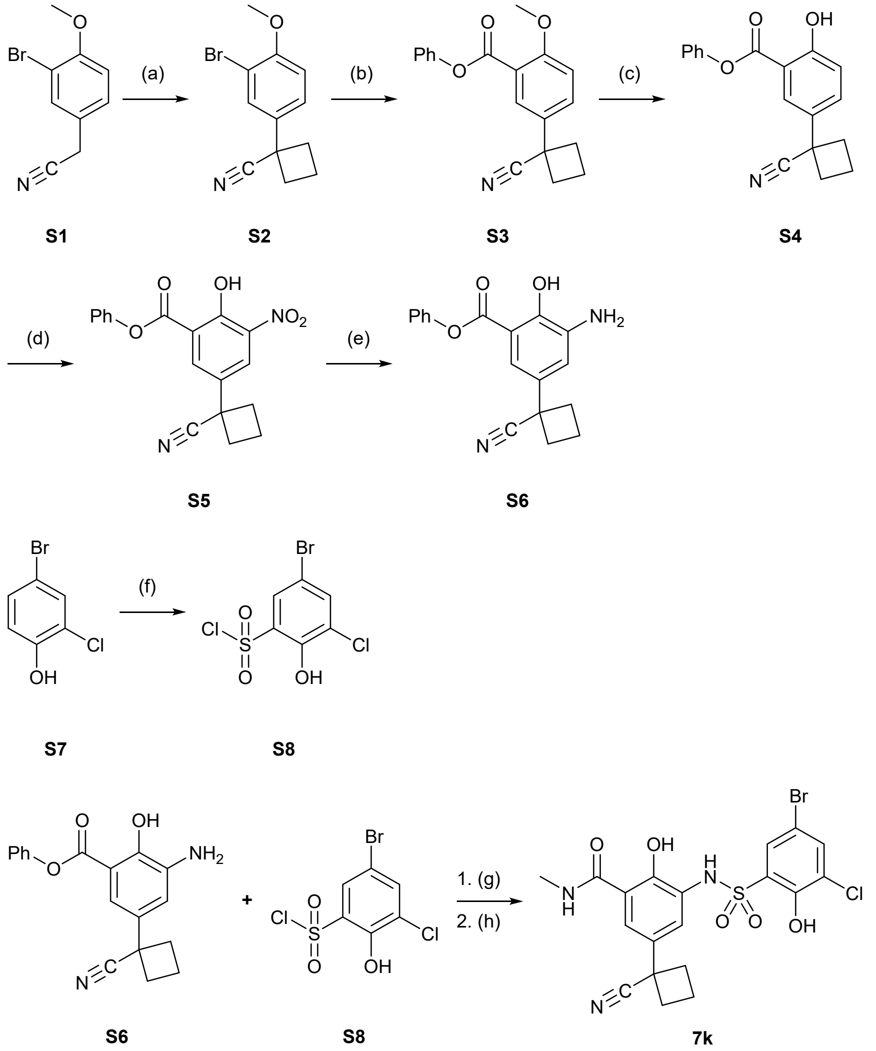

Supplemental scheme 1. Synthetic scheme for compound **2**. (a) NaH, 1,3-dibromopropane, DMSO, rt, 24 h; (b) Phenyl formate, phenol, $Pd(OAc)_2$, P(*t*-Bu)$_3$ $\cdot HBF_4$, Net_3, MeCN 90 °C, sealed tube, 24 h; (c) BBr_3, DCM, -78 °C. (d) HNO_3, TBAB, DCE/H_2O, 60 °C, 24 h; (e) H_2, Pd/C, MeOH, rt; (f) HSO_3Cl, 0 °C – rt; (g) Pyridine, DCM, rt; (h) NH_2Me, DIPEA, THF, 65 °C.

1-(3-Bromo-4-methoxyphenyl)cyclobutane-1-carbonitrile (S2)

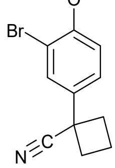

NaH (60% in mineral oil, 0.6 g, 15 mmol, 3 eq) was added portion wise to a stirring solution containing 2-(3-Bromo-4-methoxyphenyl)acetonitrile (**S1**, 1.13 g, 5 mmol, 1.0 eq) and 1,3-dibromopropane (612 µL, 6 mmol, 1.2 eq) in DMSO (50 mL). After the reaction was stirred for 16 h at rt, the reaction was quenched with EtOAc/Et_2O (1:1), then the organic phase was washed with water (3 X). The aqueous layer was back extracted with EtOAc. The organic layers were combined, washed with $brine_{(sat.)}$, concentrated, and purified by normal phase column chromatography (petroleum ether/EtOAc gradient from 19:1 to 0:1) to provide the **S2** as yellowish oil (867 mg, 3.27 mmol, 65% yield).

R_f = 0.30 (Petroleum ether: EtOAc = 9:1)

LRMS: calculated for $[M+Na]^+$ $C_{12}H_{12}{}^{79}BrNONa$: 288.0, found: 287.8

^{1}H NMR (600 MHz, Chloroform-*d*) δ 7.58 (d, *J* = 2.4 Hz, 1H), 7.32 (dd, *J* = 8.5, 2.4 Hz, 1H), 6.90 (d, *J* = 8.6 Hz, 1H), 3.91 (s, 3H), 2.84 – 2.76 (m, 2H), 2.63 – 2.53 (m, 2H), 2.48 – 2.35 (m, 1H), 2.12 – 2.00 ppm (m, 1H).

^{13}C NMR (151 MHz, Chloroform-*d*) δ 155.5, 133.3, 130.7, 125.9, 124.1, 112.2, 112.0, 56.4, 39.3, 34.8, 17.0 ppm.

Phenyl 5-(1-Cyanocyclobutyl)-2-methoxybenzoate (S3)

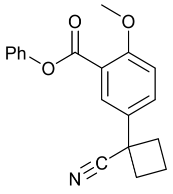

Phenyl formate (290 µL, 2.67 mmol, 2.0 eq) and NEt_3 (558 µL, 4.0 mmol, 3.0 eq) was added to a sealed tube reactor containing a solution of **S2** (335 mg, 1.34 mmol, 1.0 eq), $Pd(OAc)_2$ (15.1 mg, 0.067 mmol, 0.05 eq), (*t*-Bu)$_3$PH·BF_4 (78.4 mg, 0.26 mmol, 0.2 eq) and phenol (117 µl, 1.34 mmol, 1.0 eq) in MeCN (5 mL) with a stirring bar, then the reactor was sealed, heated to 90 ^{o}C and stirred for 24 h. After the reaction cooled down to rt, the mixture was filtered through a pad of celite, and the filtrate was collected and concentrated. The residue was redissolved in EtOAc and washed with water, then the organic layer was collected, concentrated, and purified by normal phase column chromatography (petroleum ether / EtOAc gradient from 9:1 to 0:1) to provide the product as a colorless solid (**S3**, 328.9 mg, 1.07 mmol, 80% yield).

R_f = 0.04 (Petroleum ether: EtOAc = 9:1)

LRMS: calculated for $[M+H]^+$ $C_{19}H_{18}NO_3$: 308.1, found: 308.0

¹H NMR (700 MHz, Chloroform-*d*) δ 8.02 (d, *J* = 2.6 Hz, 1H), 7.59 (dd, *J* = 8.7, 2.6 Hz, 1H), 7.45 – 7.40 (m, 2H), 7.29 – 7.26 (m, 1H), 7.25 – 7.22 (m, 2H), 7.06 (d, *J* = 8.8 Hz, 1H), 3.95 (s, 3H), 2.88 – 2.83 (m, 2H), 2.68 – 2.60 (m, 2H), 2.50 – 2.40 (m, 1H), 2.13 – 2.06 ppm (m, 1H).

¹³C NMR (176 MHz, Chloroform-*d*) δ 164.2, 159.5, 151.0, 131.8, 131.7, 129.6, 129.4, 126.0, 124.3, 122.0, 119.8, 112.9, 56.5, 39.6, 34.9, 17.2 ppm.

Phenyl 5-(1-Cyanocyclobutyl)-2-hydroxybenzoate (S4)

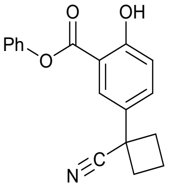

A solution of **S3** (123 mg, 0.4 mmol, 1.0 eq) in anhydrous DCM (1 ml) was cooled to – 78 °C in an acetone bath cooling with HAAKE EK90 under Argon. BBr_3 (1 M in hexane, 0.8 ml, 0.8 mmol, 2 eq) was added dropwise to the stirring solution, and the reaction was continued for another 1 h at – 78 °C, then the reaction was allowed to warm up to rt. After the reaction returned to rt, it was quenched by pouring over an ice/water slurry. The whole slurry was then extracted with EtOAc, then the organic phase was washed with water and brine successively. The organic phase was collected, dried over anhydrous $MgSO_4$, concentrated, and purified by normal phase column chromatography (petroleum ether / EtOAc gradient from 19:1 to 0:1) to provide the product as a colorless oil (89.9 mg, 0.31 mmol, 76.5% yield).

R_f = 0.26 (Petroleum ether: EtOAc = 9:1)

¹H NMR (700 MHz, Chloroform-*d*) δ 10.55 (s, 1H), 8.09 (d, *J* = 2.5 Hz, 1H), 7.59 (dd, *J* = 8.8, 2.5 Hz, 1H), 7.51 – 7.44 (m, 2H), 7.37 – 7.29 (m, 1H), 7.25 – 7.20 (m, 2H), 7.09 (d, *J* = 8.8 Hz, 1H), 2.89 – 2.83 (m, 2H), 2.68 – 2.59 (m, 2H), 2.51 – 2.41 (m, 1H), 2.14 – 2.06 ppm (m, 1H).

¹³C NMR (176 MHz, Chloroform-*d*) δ 168.7, 161.9, 150.1, 133.9, 131.1, 129.9, 127.4, 126.8, 124.3, 121.8, 118.9, 112.0, 39.6, 34.8, 17.2 ppm.

Phenyl 5-(1-Cyanocyclobutyl)-2-hydroxy-3-nitrobenzoate (S5)

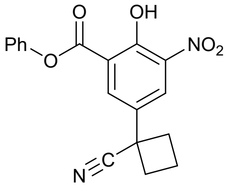

Compound **S4** (88 mg, 0.3 mmol, 1 eq) was suspended in DCE (300 µl) and the solution was cooled using an ice/water bath. A solution of Tetrabutylammonium bromide (4.83 mg, 0.015 mmol, 0.05 eq) and concentrated nitric acid (38 µl) in H_2O (300 µL) was added, followed by vigorous stirring at 60 °C for 24 h. The reaction was allowed to cool down to rt, then diluted with DCM, and the organic phase was washed with water, collected, dried over anhydrous $MgSO_4$, concentrated, and purified by normal phase column chromatography to provide the product as pale-yellow solid (71.5 mg, 0.21 mmol, 70% yield).

R_f = 0.84 (EtOAc)

LRMS: calculated for $[M+Na]^+$ $C_{18}H_{14}N_2O_5Na$: 361.1, found: 361.0

^{1}H NMR (500 MHz, Chloroform-*d*) δ 11.74 (s, 1H), 8.40 (d, *J* = 2.6 Hz, 1H), 8.29 (d, *J* = 2.5 Hz, 1H), 7.56 – 7.44 (m, 2H), 7.44 – 7.31 (m, 1H), 7.25 – 7.21 (m, 2H), 2.96 – 2.87 (m, 2H), 2.73 – 2.61 (m, 2H), 2.59 – 2.45 (m, 1H), 2.22 – 2.08 ppm (m, 1H).

^{13}C NMR (126 MHz, Chloroform-*d*) δ 167.1, 155.6, 149.7, 138.2, 133.6, 131.0, 130.0, 129.2, 127.2, 123.1, 121.5, 116.4, 39.3, 34.8, 17.2 ppm.

Phenyl 3-Amino-5-(1-cyanocyclobutyl)-2-hydroxybenzoate (S6)

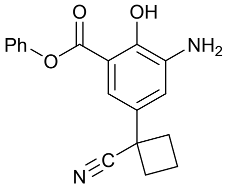

Compound **S5** (66.5 mg, 0.196 mmol, 1 eq) was added to a slurry of Pd/C (5% by wt, 10 mol%) in MeOH/EtOAc (1:1, 4 ml), then the hydrogenation was carried out by stirring under H_2 atmosphere for 16 h. After the starting material was consumed (monitored by TLC), the reaction was filtered through a pad of celite, the filtrate was collected, concentrated and purified by normal phase column chromatography (petroleum ether / DCM gradient from 1:1 to 0:1, then DCM / EtOAc gradient from 9:1 to 0:1) to give the product (27.4 mg, 0.09 mmol, 45%).

R_f = 0.19 (DCM)

LRMS: calculated for $[M+H]^+$ $C_{18}H_{17}N_2O_3$: 309.1, found: 309.0

^{1}H NMR (500 MHz, DMSO-d_6) δ 10.94 – 9.55 (br, s, 1H), 7.53 – 7.45 (m, 2H), 7.38 – 7.30 (m, 3H), 7.22 (d, *J* = 2.3 Hz, 1H), 7.01 (d, *J* = 2.4 Hz, 1H), 6.00 – 4.65 (br, s, 2H), 2.75 – 2.65 (m, 2H), 2.62 – 2.53 (m, 2H), 2.33 – 2.19 (m, 1H), 2.07 – 1.93 (m, 1H).

^{13}**C NMR** (126 MHz, DMSO-d_6) δ 168.3, 150.0, 148.1, 138.8, 130.9, 129.7, 126.4, 124.6, 122.1, 115.4, 112.6, 110.8, 39.7, 33.7, 16.7.

5-Bromo-3-chloro-2-hydroxybenzenesulfonyl Chloride (S8)

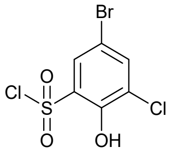

Chlorosulfonic acid (932 µl, 14 mmol, 7 eq) was cooled down to 0°C. 4-bromo-2-chlorophenol (**S7**, 514 mg, 2.48 mmol, 1 eq) was added portion-wise and stirred for 1 h at 0°C. After warming to room temperature and stirring for 23 h, the reaction was quenched by carefully pouring over a slurry of ice, DCM, and brine. The whole slurry was extracted with DCM, and the organic layer was collected, dried over anhydrous $MgSO_4$, concentrated then purified by normal phase column chromatography (petroleum ether / DCM gradient from 1:1 to 0:1) to provide the pure product as white crystals (560 mg, 1.83 mmol, 73% yield).

R_f = 0.89 (DCM)

^{1}**H NMR** (500 MHz, DMSO-d_6) δ 11.94 (s, 1H), 7.64 (d, *J* = 2.4 Hz, 1H), 7.48 ppm (d, *J* = 2.4 Hz, 1H).

^{13}**C NMR** (126 MHz, DMSO-d_6) δ 148.9, 133.5, 133.0, 128.2, 121.8, 109.2 ppm.

3-((5-Bromo-3-chloro-2-hydroxyphenyl)sulfonamido)-5-(1-cyanocyclobutyl)-2-hydroxy-N-methylbenzamide (7k)

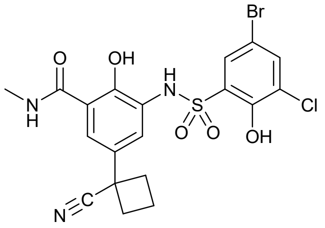

S8 (33.6 mg, 110 µmol, 1.2 eq) and pyridine (21.8 µl, 270 µmol, 3 eq) were added to compound **S6** (27.4 mg, 90 µmol, 1 eq) in DCM (0.45 ml). The resulting reaction mixture was stirred for 5 h at room temperature. Additional compound **S8** (16.8 mg, 55 µmol, 0.6 eq) and pyridine (7.3 µl, 90 µmol, 1 eq) were added and the mixture was stirred for another 2 h at room temperature. The mixture was concentrated under reduced pressure, diluted with

EtOAc, and washed with 0.1 M $HCl_{(aq)}$ and brine. The organic layer was collected, dried over anhydrous $MgSO_4$, concentrated under reduced pressure, then purified by normal phase column chromatography (MeOH / DCM gradient from 0:1 to 3:17) to give the intermediate product **S9** (20 mg, 35 µmol, 35% yield).

To a solution of Compound **S9** (20 mg, 35 µmol, 1 eq) in THF (1 mL) was added NH_2Me (2 M in methanol, 86 µL, 173 µmol, 5 eq) in one portion. The reaction was sealed and stirred at rt for 1 h. The reaction was concentrated, then purified by preparative HPLC (0.1% TFA in H_2O / 0.1% TFA in ACN gradient from 18:7 to 1:49) to give the product **S10** (6.49 mg, 12.6 µmol, 37% yield).

Retention time by analytical HPLC: 15.21 min

Purity of product by analytical HPLC: 98.0% (UV 254 nm)

HRMS: calculated for $[M+H]^+$ $C_{19}H_{18}O_5N_3{}^{79}Br^{37}ClS$: 515.98041, found: 515.98074

^{1}H NMR (700 MHz, DMSO-d_6) δ 13.75 (s, 1H), 11.09 (s, 1H), 9.39 (s, 1H), 9.12 (d, *J* = 4.5 Hz, 1H), 7.93 (d, *J* = 2.5 Hz, 1H), 7.71 (d, *J* = 2.2 Hz, 1H), 7.71 (d, *J* = 2.5 Hz, 1H), 7.35 (d, *J* = 2.2 Hz, 1H), 2.82 (d, *J* = 4.6 Hz, 3H), 2.72 – 2.66 (m, 2H), 2.55 – 2.51 (m, 2H), 2.29 – 2.20 (m, 1H), 2.00 – 1.92 (m, 1H).

^{13}C NMR (176 MHz, DMSO-d_6) δ 169.5, 153.4, 150.5, 136.5, 130.3, 129.6, 129.2, 125.6, 124.3, 124.09, 123.98, 120.8, 114.5, 109.5, 38.9, 33.7, 26.0, 16.6 ppm.

Preparation of peptides

In general, peptides were synthesized through the conventional Fmoc strategy. Rink Amide AM resin (0.3 – 0.6 mmol/g) was used as solid support. The reactions were conducted at room temperature unless otherwise specified. Isolated yields were calculated by dividing the amount of purified product by the amount of resin loading.

Synthesis of WIN peptide

Peptide synthesis was performed using an automated Syro I parallel peptide synthesizer. Fmoc was deprotected by treating the resin with 25% Piperidine in DMF for 3 min twice. The new amino acid was coupled by treating resin with 4 eq amino acids, 4 eq PyBOP, and 8 eq DIPEA in DMF for 1 h. Ac capping was performed by treating resin with 10 eq Ac_2O, and 10 eq DIPEA in DMF for 15 min.

Synthesis of FAM-labeled WIN peptide (**1F**)

Ac—A-R-T-E-V-H-L-R-K-S—NH_2

Fmoc-Lys(Alloc)-OH was used as an orthogonally protected residue that could be selectively deprotected for FAM labeling. After the linear sequence was synthesized by the synthesizer, the Fmoc on the N-terminus was removed and capped with Ac_2O (10 eq) and DIPEA (10 eq) in DMF for 30 min. The alloc group on the lysine was deprotected by treating resin with 0.25 eq $Pd(PPh_3)_4$, 25 eq $PhSiH_3$ in DCM for 30 min twice[1], then the resin was washed with 0.5% sodium diethyldithiocarbamate in DMF for 5 min and a total of five times. FAM labeling was carried out by treating the resin with 5-FAM (2 eq), PyBOP (2 eq), and DIPEA (3 eq) in DMF for 24 h.

After the sequence was ready, peptides were cleaved from the resin by treating the resin in a solution of TFA/H_2O/TIPS (95/2.5/2.5) for 1 h. After the cleavage was done, the resin was filtered off and the filtrate was concentrated by blowing N_2 over the surface followed by precipitation of the peptide in cold diethyl ether three times. The product was then purified by preparative HPLC equipped with a Macherey-Nagel column (5 µm, 125 x 21 mm, flow rate: 20 mL/min), eluting with a linear gradient from 10% to 60% (percentage of B. A: 0.1% TFA in H_2O; B: 0.1% TFA in ACN) over 60 min. The elution was monitored at 254 nm. The pure fractions were pooled together and lyophilized to give the desired product (3.1 mg, 3.1%).

Retention time by analytical HPLC: 8.607 min

Purity of product by analytical HPLC: 98.6% (UV 254 nm)

HRMS: calculated for $[M+2H]^{+2}$ $C_{91}H_{138}O_{24}N_{24}$: 975.51524, found: 975.51661

Synthesis of non-labeled WIN peptide (**1NH$_2$**)

Ac—A–R–T–E–V–H–L–R–K–S—NH$_2$

After the linear sequence was synthesized by the synthesizer, the Fmoc on the N-terminus was removed and capped with Ac_2O (10 eq.) and DIPEA (10 eq.) in DMF for 30 min.

After the sequence was ready, peptides were cleaved from the resin by treating the resin in a solution of TFA/H_2O/TIPS (95/2.5/2.5) for 1 h. After the cleavage was done, the resin was filtered off and the filtrate was concentrated by blowing N_2 over the surface followed by precipitation of the peptide in cold diethyl ether three times. The product was then purified by preparative HPLC equipped with a Macherey-Nagel column (5 µm, 125 x 21 mm, flow rate: 20 mL/min), eluting with a linear gradient from 10% to 60% (percentage of B. A: 0.1% TFA in H_2O; B: 0.1% TFA in ACN) over 60 min. The elution was monitored at 210 nm. The pure fractions were pooled together and lyophilized to give the desired product (53.1 mg, 43% yield).

Retention time on analytical HPLC: 6.322 min

Purity of product on analytical HPLC: 96.7% (UV 210 nm)

HRMS: calculated for $[M+2H]^{+2}$ $C_{52}H_{94}O_{15}N_{20}$: 619.35983, found: 619.35941

Synthesis cMYC-cyclic peptide

The peptide was synthesized using the same protocol as described in our previous publication[2]. Peptide synthesis was performed using an automated PurePep Chorus parallel peptide synthesizer. Fmoc was deprotected by treating resin with 25% Piperidine in DMF for 3 min twice at 50 ^{o}C. Amino acids were coupled by treating resin with 5 eq amino acid, 5 eq HCTU, and 10 eq DIPEA in DMF for 10 min at 75 ^{o}C. Ac capping was performed by treating the resin with 10 eq Ac_2O, and 10 eq DIPEA in DMF for 10 min.

Allyl/Alloc protecting groups were used for selective side chain protection. The elongation of peptides was paused when the second amino acid position for cyclization was reached. An *o*-Ns protecting strategy was used to prevent premature N-terminal Fmoc deprotection during cyclization. Fmoc was deprotected with 25% piperidine in DMF for 10 min twice. Reprotection was performed by treating resin in 4 eq *o*-NsCl, 5 eq 2,4,6-collidine, and 5 eq DMAP in DMF for 15 min twice[2,3]. The side chain was deprotected by treating resin with 0.1 eq $Pd(PPh_3)_4$, 0.4 eq PPh_3, and 8 eq Pyrrolidine in DCM for 1 h[2,4]. Then the resin was washed with 0.5% sodium diethyldithiocarbamate in DMF for 5 min and a total of five times. The carboxylic group on the side chain was activated by treating the resin with 2 eq PyAOP, and 2 eq HOAt in DMF for 15 min, then 4 eq 2,4,6-collidine was added directly to the reaction and continued the reaction for 48 h. Capping of the unreacted side chain was carried out by treating the resin in 10 eq Ac_2O, and 10 eq DIPEA in DMF for 30 min. *o*-Ns group was deprotected by treating resin with 10 eq 2-mercaptoethanol and 5 eq of DBU in DMF for 16 h. Sequence elongation was then continued after cyclization and *o*-Ns deprotection were done.

Synthesis of FITC-labeled cMYC-cyclic peptide (**2F**)

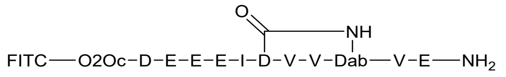

Sequences were further elongated with Fmoc-O2Oc-OH after the sequence was done. Fmoc was deprotected with 25% Piperidine in DMF for 10 min twice. FITC labeling was performed by treating resin with 2 eq 6-FITC, 4 eq DIPEA in DMF for 16 h.

After the sequence was ready, peptides were cleaved from the resin by treating the resin in a solution of TFA/H_2O/TIPS (95/2.5/2.5) for 1 h. After the cleavage was done, the resin was filtered off and the filtrate was concentrated by blowing N_2 over the surface followed by precipitation of the peptide in cold diethyl ether three times. The product was then purified by preparative HPLC equipped with a Macherey-Nagel column (5 µm, 125 x 21 mm, flow rate: 20 mL/min), eluting with a linear gradient from 5% to 60% (percentage of B. A: 15 mM $NH_4HCO_{3(aq.)}$; B: MeOH) over 60 min. The elution was monitored at 254 nm. The pure fractions were pooled together and lyophilized to give the desired products (28 mg, 7.8%).

Retention time on analytical HPLC: 10.70 min

Purity of product on analytical HPLC: 96.0% (UV 254 nm)

HRMS: calculated for $[M+H]^+$ $C_{80}H_{108}O_{30}N_{15}S$: 1790.70992, found: 1790.71017

Synthesis of none-labeled cMYC-cyclic peptide (**2Ac**)

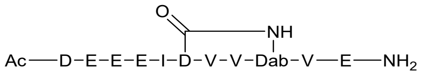

After the sequence was fully elongated, Fmoc was deprotected with 25% Piperidine in DMF for 10 min twice. Ac capping was performed by treating resin with 10 eq Ac_2O, 10 eq DIPEA and DMF for 30 min.

After the sequence was ready, peptides were cleaved from the resin by treating the resin in a solution of TFA/H_2O/TIPS (95/2.5/2.5) for 1 h. After the cleavage was done, the resin was filtered off and the filtrate was concentrated by blowing N_2 over the surface followed by precipitation of the peptide in cold diethyl ether three times. The product was then purified by preparative HPLC equipped with a Macherey-Nagel column (5 µm, 125 x 21 mm, flow rate: 20 mL/min), eluting with a linear gradient from 5% to 60% (percentage of B. A: 15 mM $NH_4HCO_{3(aq.)}$; B: MeOH) over 60 min. The elution was monitored at 210 nm. The pure fractions were pooled together and lyophilized to give the desired products (8.2 mg, 12.5%).

Retention time on analytical HPLC: 8.746 min

Purity of product on analytical HPLC: >99.5% (UV 210 nm)

HRMS: calculated for $[M+2H]^{+2}$ $C_{55}H_{89}O_{23}N_{13}$: 649.80916, found: 649.80998

Recombinant protein expression and purification

WDR5^{WT}-FLAG (Δ1-21) and WDR5^{F266A}-FLAG (Δ1-21) were expressed in BL21 DE RIL *Escherichia coli*. *E. coli* was grown in LB medium supplemented with 0.1% ampicillin and the expression was induced with 200 µM isopropyl-beta-D-thiogalactopyranoside (IPTG) at 20 °C for 16 h. After centrifugation, the pellets were resuspended in Buffer A additionally supplemented with 1 mM PMSF and a spatula tip of DNase I (Roche, #04716728001), then disrupted using a sonicator CL-334 (70% amplitude, 10 s pulse with 6 min interval) while the solution was kept cold by placing it on ice. The insoluble cell components were pelleted by centrifugation at 60000 rcf under 4 °C for 45 min. The supernatant containing the desired protein was filtered with a 0.22 µm syringe filter, loaded on HisTrap HP His tag protein

purification column (Cytiva, #17524801) with Buffer A and eluted by a linear gradient to Buffer B (100%, 100 min). The desired fractions were collected, pooled together, and dialysis with Prescission-3C-protease (3.5 mg/mL) at 4 °C for 16 h. The dialyzed protein was then purified by reverse Ni-NTA with a linear gradient of Buffer A to Buffer B in 50 min and concentrated with an Amicon® ultra centrifugation filter (4000 rcf, 10 min, 4 °C). The concentrated protein was cleared by centrifugation (10 min, 4 °C, 20000 rcf), and the supernatant was loaded directly on a Superdex 75 16/600 column eluting with SEC buffer (isocratic, flow = 1 mL/min). For each purification step, the protein of interest was analyzed by 15% SDS-Page polyacrylamide gels and staining by Coomassie Blue staining solution.

Fluorescence Polarization

Fluorescence polarization was measured using a Tecan Spark Microplate Reader with 384-well plates (Corning, #4514). The assay buffer contained 25 mM HEPES, 150 mM NaCl, 0.01% Triton-X and 1% DMSO at pH 7.0. Monochromator was applied for the fluorescence measurement and the excitation wavelength was set to 485 nm with a bandwidth of 20 nm, while the emission wavelength was set to 535 nm with a bandwidth of 20 nm. All FP experiments were performed as two biological replicates of two technical replicates each.

FP measurement for direct binding assay

A 15-point dose-response curve was generated for direct binding assays by titrating a twofold dilution series of protein against fixed FITC-labelled peptide (0.6 nM or 5 nM). A sample of protein-free corresponding FITC-labelled peptide in the assay buffer was included as non-binding control for each set of replicates. After the plate was prepared, it was sealed and incubated at 25 °C for 1 h before measurement. The raw value of fluorescence polarization was used, and K_D (dissociation constant) was calculated by fitting with GraphPad Prism 9 using the [Agonist] vs. Response – variable slope (four parameter) function for curve fitting.

Supplemental Table 1. Concentration of the components in FP direct binding assay.

Condition	Starting protein concentration (two-fold dilution)	Probe concentration (Fixed concentration)
FLAG-WDR5^{WT}-WIN	1 µM	0.6 nM (**1F**)
FLAG-WDR5^{WT}-WBM	40 µM	5.0 nM (**2F**)
FLAG-WDR5^{F266A}-WIN	1 µM	0.6 nM (**1F**)
FLAG-WDR5^{F266A}-WBM	40 µM	5.0 nM (**2F**)

FP measurement for competitive assay

Competitive fluorescence polarization measurements were performed by 2-fold dilution series of the inhibitor against a fixed concentration of WDR5 and **tracer**. The result was

plotted using a logarithmic x-axis and normalized using the theoretical maximum and minimum binding values as 100 and 0% respectively. The raw value of fluorescence polarization was used, and IC_{50} was calculated by fitting with log(Inhibitor) vs. normalized response – variable slope function for curve fitting of GraphPad Prism for each individual replicate. The IC50 values was then converted to the K_I by the method reported by Nikolovska-Coleska *et al*[5]. using the following equation:

$$K_I = [I]_{50}/(\frac{[L]_{50}}{K_D} + \frac{[P]_0}{K_D} + 1)$$

In this equation, $[I]_{50}$ is the concentration of the unbound inhibitor at 50% inhibition, $[L]_{50}$ is the concentration of the unbound tracer at 50% inhibition, $[P]_0$ refers to the concentration of the free protein without any inhibition and K_D is the affinity between tracer and protein. The calculations were made using the excel sheet provided by the authors which can be found at: http://websites.umich.edu/~shaomengwanglab/software/calc_ki/index.html

After all Ki values were calculated for a single compound they were averaged and the error was calculated as the standard deviation.

Supplemental Table 2. Concentration of the components in competitive FP binding assay.

Testing site	Inhibitor	Inhibitor conc. (two-fold dilution)	Protein conc. ($WDR5^{WT}$, fixed)	Probe conc. (fixed)
WIN	**$1NH_2$**	5 µM	2 nM	0.6 nM (**1F**)
WIN	**OICR-9429**	1 µM	2 nM	0.6 nM (**1F**)
WIN	**2Ac**	40 µM	2 nM	0.6 nM (**1F**)
WIN	**7k**	40 µM	2 nM	0.6 nM (**1F**)
WBM	**$1NH_2$**	40 µM	500 nM	5.0 nM (**2F**)
WBM	**OICR-9429**	40 µM	500 nM	5.0 nM (**2F**)
WBM	**2Ac**	40 µM	500 nM	5.0 nM (**2F**)
WBM	**7k**	40 µM	500 nM	5.0 nM (**2F**)

Cell line culturing

U-2 OS (DSMZ, ACC 785) was grown in DMEM high glucose medium (Sigma-Aldrich, #D6429) supplemented with 10% Fetal Bovine Serum (FBS, Cellsera, #AU-FBSPG) and 1% Penicillin-Streptomycin (Pen-Strep, Gibco, #15140-122) in a humidified atmosphere with 5% CO_2 under 37 ^{o}C. The subcultivation ratio followed the suggestion from ATCC.

MDA-MB-231 (DSMZ, ACC 732) were grown in DMEM, high glucose, GlutaMAX supplement, pyruvate (Gibco, 31966-021) supplemented with 10% Fetal Bovine Serum (FBS, Cellsera, #AU-FBSPG) and 1% Penicillin-Streptomycin (Pen-Strep, Gibco, #15140-122) in a humidified atmosphere with 5% CO_2 under 37 ^{o}C. The subcultivation ratio followed the suggestion from ATCC.

Total RNA isolation

For isolation of total RNA, cells in a Petri dish were washed with PBS (Gibco, #10010-023), trypsinized (0.05% Trypsin, Gibco, #25300-054), followed by pelleting of the cells by centrifugation (3 min, rt, 200 rcf). The pellet was then washed with PBS, resuspended in TRIzol (Invitrogen, #15596026), and the protocol from the manufacturer was followed to obtain total RNA isolates.

RNA immunoprecipitation (RNA-IP)

Total RNA isolates, purified proteins, and inhibitors were obtained as previously described. The protocol was adapted from Fuentes-Iglesias *et al*[6]. 10 µL of ANTI-FLAG® M2 Magnetic Beads (Millipore, #M8823) were washed with RIP Buffer C, then loaded with target protein (0.2 mg/mL, in Buffer C) supplemented with 0.1% IGEPAL, 0.1% BSA (Serva, #11930.03) and 10 µg/mL yeast tRNA (Biosciences, 058Y) at 4 ^{o}C for 3 h. After the incubation, the beads were washed with 0.1% IGEPAL in Buffer C five times. 1 µg of total RNA isolated from U-2 OS or MDA-MB-231, w/wo inhibitor, and 0.1% DMSO in RNA-immunoprecipitation buffer were incubated with protein-beads complex at rt for 1 h. After the incubation, the beads were washed with RNA-immunoprecipitation buffer 5 times and then rinsed with 50 µL of RNase free water once. The RNA/protein bound beads were resuspended in 50 µL of RNase free water. From this resuspended solution, 5 µL was transferred and mixed with 2X Laemmli buffer, loaded on a 15% SDS-PAGE polyacrylamide gel to analyze the protein loading of each sample. The other 45 µL was treated with 450 µL of TRIzol following the TRIzol protocol to extract the RNA. After the extraction process, the RNA pellet was dissolved in 10 µL of RNase free water and used for RT-qPCR analysis. A sample of 0.5 µg RNA input underwent the same treatment starting from resuspension in 50 µL RNAse free water, including sampling for gel and TRIzol extraction, to control for sample loss between RIP product and non-treated RNA input.

lncRNA expression level assay

MDA-MB-231 cells were seeded (1 x 10^5 cells/well for one-day treatment, or 5 x 10^4 cells/well for three-day treatment.) in a Biolite 12 well Multidish (Thermo Scientific, 130185) for one day (37 ^{o}C, 5% CO_2). After the cells were settled, the selected compound concentration or blank (DMSO 0.1%) was prepared in the fresh normal growing medium. Then the old growing medium was changed into the selected medium and incubated at 37 ^{o}C, 5% CO_2 for the desired incubation time.

Quantitative reverse transcription-polymerase chain reaction (RT-qPCR)

cDNA was synthesized by using high-capacity cDNA reverse transcription kit (Applied Biosystems, 4368814) following the manufacturer's protocol. For RNA from RIP, all the RNA for each sample was directly put in the reaction with a reaction volume of 20 µL. For samples originating from cell treatment, the concentration of RNA was determined by nanodrop, then 0.5 µg of RNA was put in 20 µL of reaction to generate the cDNA. After cDNA was generated, it was analyzed by PowerUp™ SYBR™ Green Master Mix (Applied Bioscience, #A25742) via the fast-cycling mode and supplemented with 500 nM for both forward and reverse primer according to the target amplicon.

Data analysis

Percentage of enrichment for RNA-IP

Percentage of enrichment was calculated by normalization with 0.5 µg (50%) of RNA input. The percentage of enrichment was calculated by the method reported by Fuentes-Iglesias *et al*[6]. In short, the difference of Ct between the sample after enrichment and the 0.5 µg of RNA input (compensated with the percentage of dilution, 50%) was calculated.

$$Ct_{sample} - (Ct_{input} - \log_2{(1/percentage\ of\ dilution)}) = \Delta Ct_{corrected}$$

Then the percentage of target RNA enrichment from the RNA pool can be calculated by the following equation:

$$Percentage\ of\ enrichment = 2^{-\Delta Ct_{corrected}} \times 100\%$$

The student t-test was carried out by putting the average of mean (Mean) and the standard deviation (SD) with number of replicates (N) into GraphPad prism 9 and calculated from multiple unpaired t-tests with no assumption about consistent SDs (Welch t-test) for each set of comparisons.

Fold change of gene expression level

The raw Ct value observed from the qPCR instrument was used in $2^{-\Delta\Delta Ct}$ method to calculate the fold change of target gene expression level and GAPDH was used as the internal reference gene[7]. In short, the expression level of target gene was first normalized to GAPDH, then normalized to the expression level of the blank to give $\Delta\Delta Ct_{sample}^{gene}$.

$$Ct^{gene} - Ct^{GAPDH} = \Delta Ct^{gene}$$

$$\Delta Ct_{sample}^{gene} - \Delta Ct_{blank}^{gene} = \Delta\Delta Ct_{sample}^{gene}$$

Then the fold change was calculated from the following equation:

$$Fold\ change = 2^{-\Delta\Delta Ct_{sample}^{gene}} \times 100\%$$

Each data point was represented by the mean of individual bioreplicates, and the standard deviation was calculated from the group of mean by Graphpad prism 9 automatically. The significance of the difference of each sample to the reference was calculated by Graphpad prism 9 using the one sample student t-test and the target value was set to one (reference was normalized to one), and the significance of the difference between samples was calculated by Graphpad prism 9 using t-test with settings of Welcher's corrections (unpaired, two-tailed).

Appendix

Buffer recipe list:

Buffer A:

50 mM HEPES, 300 mM NaCl, 30 mM Imidazole, 0.1 mM PMSF, 1 mM TCEP, pH = 8.0

Buffer B:

50 mM HEPES, 300 mM NaCl, 500 mM Imidazole, 1 mM TCEP, pH = 8.0

Buffer C:

25 mM HEPES, 150 mM NaCl, pH = 7.0

RNA-immunoprecipitation buffer[6]:

25 mM Tris-HCl, 150 mM KCl, 5 mM EDTA, 0.1% IGEPAL, 1 mg/mL BSA, 10 µg/mL yeast tRNA, 80 U/mL Recombinant RNasin Ribonuclease Inhibitor (freshly added, Promega, #N251A), pH 7.5

SEC Buffer:

50 mM HEPES, 300 mM NaCl, 1 mM TCEP, pH = 8.0

FP assay buffer:

25 mM HEPES, 150 mM NaCl, 0.01% Triton-X and 1% DMSO, pH 7.0

Primer List

GAPDH_Fw	AGCCACATCGCTCAGACAC[8]
GAPDH_Rev	GCCCAATACGACCAAATCC[8]
U6_Fw	CTCGCTTCGGCAGCACATATAC[9]
U6_Rev	GGAACGCTTCACGAATTTGC[9]
HOTTIP_Fw	TACCGGAATAGTGCTGGGGA[10]
HOTTIP_Rev	TGCGTGCTGCTCTGAGTTTA[10]
HOXC13-AS_Fw	GAAACTGCATTTCCTGGGGC[11]
HOXC13-AS_Rev	GGCTGGAGTCTTTGTCCTCC[11]
FOXD3-AS1_Fw	GCGAGGATGTGTGGCCAAT[11]
FOXD3-AS1_Rev	AGACAGGGATTGGGTTCCGT[11]

TGF-β1_Fw CGTGGAGCTGTACCAGAAATAC[12]

TGF-β1_Rev CACAACTCCGGTGACATCAA[12]

HPLC analysis of compounds

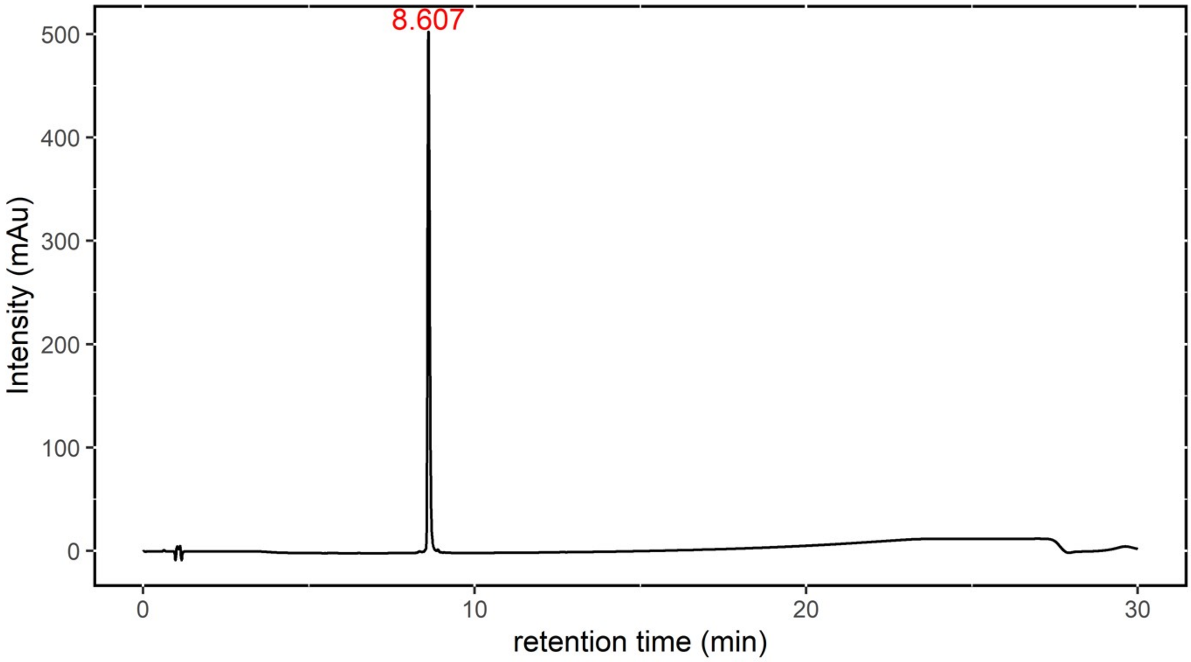

Supplemental Figure 1: HPLC analysis of **1F** (UV 254 nm)

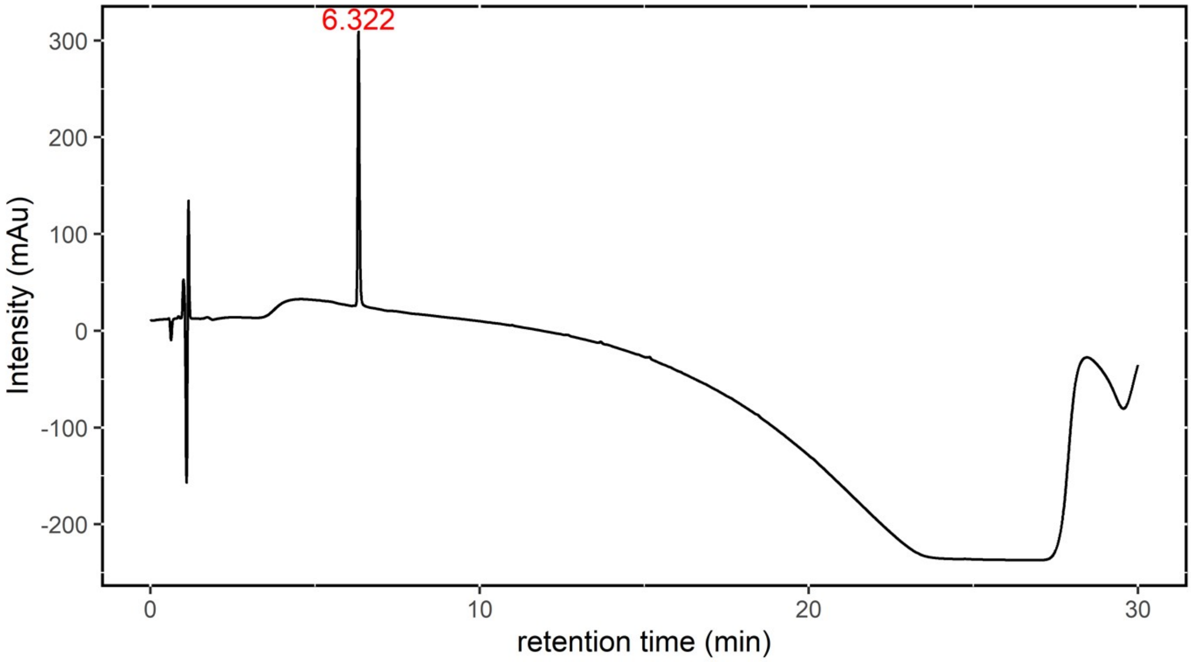

Supplemental Figure 2: HPLC analysis of **1Ac** (UV 210 nm)

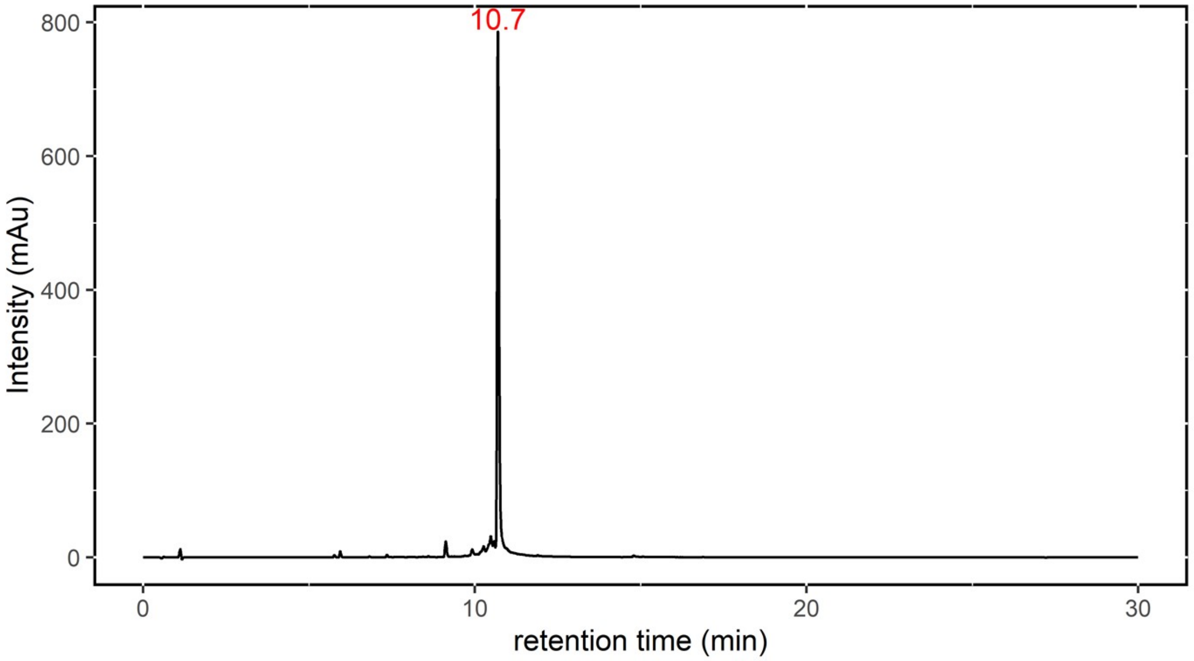

Supplemental Figure 3: HPLC analysis of **2F** (UV 254 nm)

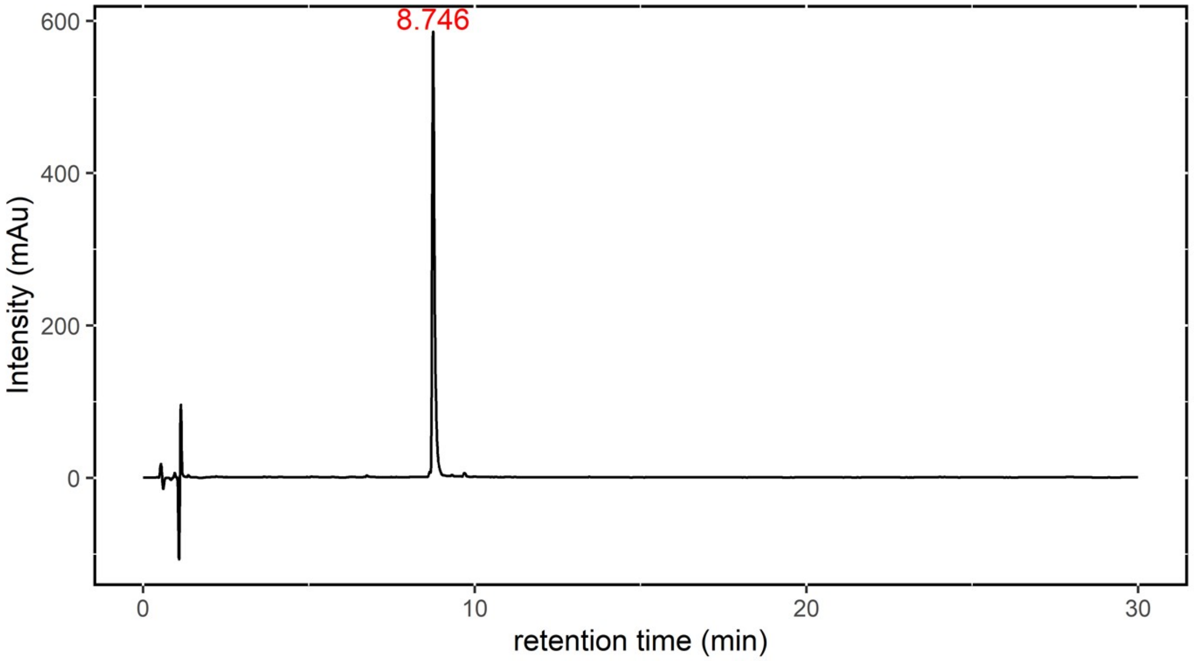

Supplemental Figure 4: HPLC analysis of **2Ac** (UV 210 nm)

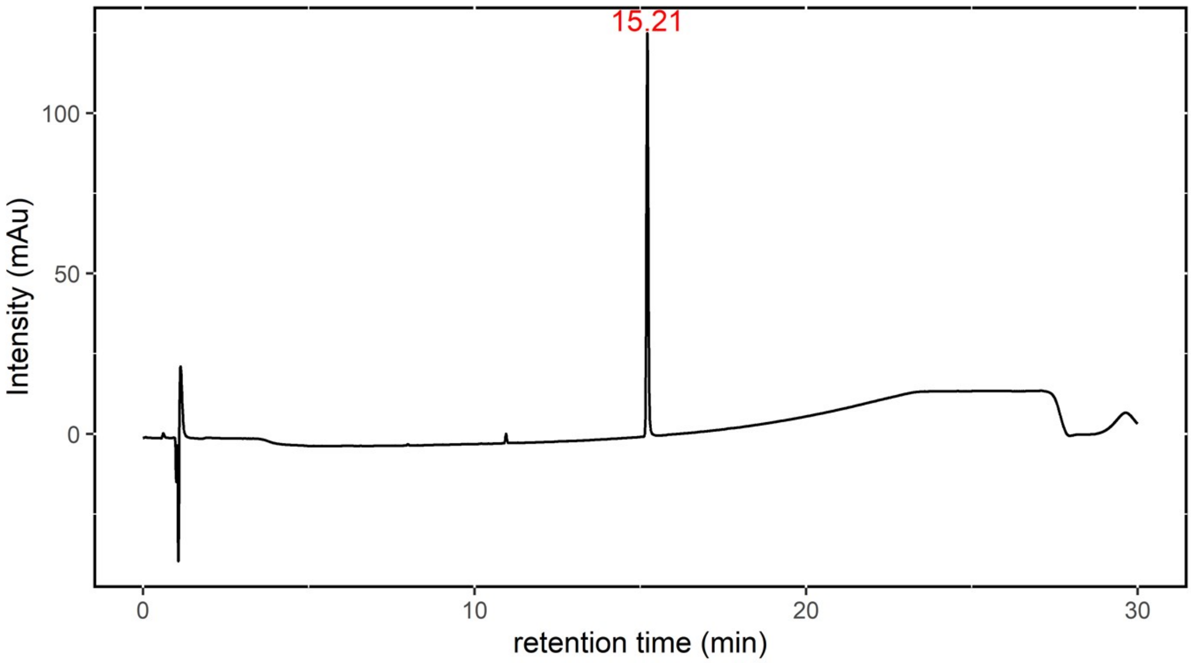

Supplemental Figure 5: HPLC analysis of molecule **7k** (UV 254 nm)

Normal lncRNA expression level in different cell lines

Supplemental Table 3. Normal Ct value of the selected lncRNA in the cell line without any treatment.

Cell line	U-2 OS	MDA-MB-231
Ct of GAPDH (Mean ± SD)	15.43 ± 0.15	15.66 ± 0.11
Ct of U6 (Mean ± SD)	19.62 ± 0.04	19.44 ± 0.12
Ct of HOTTIP (Mean ± SD)	27.82 ± 0.19	29.70 ± 0.41
Ct of HOXC13-AS (Mean ± SD)	24.30 ± 0.08	n.d.
Ct of FOXD3-AS1 (Mean ± SD)	n.d.	27.54 ± 0.10

n.d.: not detected.

Results of RIP

Supplemental Table 4. Values and details of *iv*-RIP in Figure 3.

RNA of origin	U-2 OS					
Sample name	DMSO	**OICR-9429**	**7k**	WDR5^{F266A}	No RNA	GFP
Sample label	0.1%	1 µM	5 µM	0.5 µg	10 µM	10 µM
Percentage of HOTTIP enrichment (mean ± sd)	21.67 ± 2.47	17.97 ± 2.88	13.29 ± 1.87	10.81 ± 1.53	n.d.	n.d.
Percentage of HOXC13-AS enrichment (mean ± sd)	19.32 ± 2.74	14.99 ± 1.57	13.39 ± 1.23	8.31 ± 1.91	n.d.	n.d.
Number of replicates	3	3	3	3	3	3

n.d.: not detected.

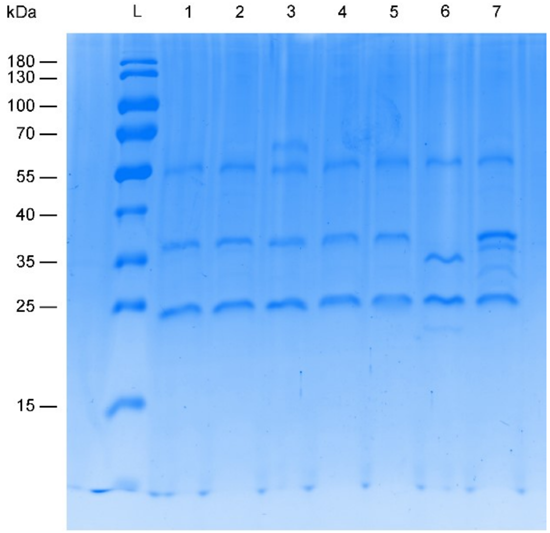

Nr	L	1	2	3	4	5	6	7
Label	Ladder	Water Blank	DMSO Blank	**OICR-9429**	**7k**	No RNA	FLAG-GFP	WDR5^{F266A}
Protein	-	FLAG-WDR5	FLAG-WDR5	FLAG-WDR5	FLAG-WDR5	FLAG-WDR5	FLAG-GFP	FLAG-F266A
RNA	-	+	+	+	+	-	-	+
Molecule	-	-	-	**OICR-9429** (10 µM)	**7k** (10 µM)	-	-	-

Supplemental Figure 6. Protein loading control of RIP was analyzed by 15% SDS-Page polyacrylamide gels. Gel was stained by Coomassie Blue staining solution to visualize the protein loading.

Results of cell treatment

Supplemental Table 5. Raw value of fold change observed from one-day treatment in each individual bioreplicates.

Exp. Nr.	Condition	Conc.	Time	HOTTIP	FOXD3-AS1
				Mean ± SD	Mean ± SD
1	Blank	0 µM	1 d	1.000 ± 0.279	1.000 ± 0.116
	**OICR-4929**	10 µM	1 d	0.920 ± 0.229	0.769 ± 0.075
	**7k**	10 µM	1 d	0.963 ± 0.284	0.913 ± 0.098
2	Blank	0 µM	1 d	1.000 ± 0.145	1.000 ± 0.022
	**OICR-4929**	10 µM	1 d	0.704 ± 0.183	0.625 ± 0.035
	**7k**	10 µM	1 d	1.521 ± 0.182	1.000 ± 0.050
3	Blank	0 µM	1 d	1.000 ± 0.161	1.000 ± 0.171
	**OICR-4929**	10 µM	1 d	1.078 ± 0.174	0.889 ± 0.111
	**7k**	10 µM	1 d	1.129 ± 0.176	0.842 ± 0.122
4	Blank	0 µM	1 d	1.000 ± 0.228	1.000 ± 0.051
	**OICR-4929**	10 µM	1 d	0.709 ± 0.128	0.693 ± 0.048
	**7k**	10 µM	1 d	0.970 ± 0.197	0.826 ± 0.055
5	Blank	0 µM	1 d	1.000 ± 0.189	1.000 ± 0.043
	**OICR-4929**	10 µM	1 d	0.793 ± 0.111	0.841 ± 0.041
	**7k**	10 µM	1 d	0.988 ± 0.141	1.053 ± 0.047
6	Blank	0 µM	1 d	1.000 ± 0.015	1.000 ± 0.037
	**OICR-4929**	10 µM	1 d	0.607 ± 0.020	0.780 ± 0.071
	**7k**	10 µM	1 d	0.618 ± 0.071	0.901 ± 0.056

Supplemental Table 6. Raw value of fold change observed from three-day treatment for each individual bioreplicate.

Exp. Nr.	condition	conc.	time	HOTTIP	FOXD3-AS1
				Mean ± SD	Mean ± SD
7[a]	Blank	0 µM	3 d	1.000 ± 0.124	1.000 ± 0.106
	**OICR-4929**	10 µM	3 d	0.680 ± 0.110	0.537 ± 0.055
	**7k**	10 µM	3 d	0.464 ± 0.082	0.756 ± 0.076
8[a]	Blank	0 µM	3 d	1.000 ± 0.023	1.000 ± 0.090
	**OICR-4929**	10 µM	3 d	0.463 ± 0.056	0.693 ± 0.059
	**7k**	10 µM	3 d	0.570 ± 0.035	1.075 ± 0.124
9[a]	Blank	0 µM	3 d	1.000 ± 0.160	1.000 ± 0.095
	**OICR-4929**	10 µM	3 d	0.528 ± 0.080	0.615 ± 0.065
	**7k**	10 µM	3 d	0.733 ± 0.095	0.847 ± 0.077
10	Blank	0 µM	3 d	1.000 ± 0.092	1.000 ± 0.049
	**OICR-4929**	10 µM	3 d	0.453 ± 0.056	0.800 ± 0.056
	**7k**	10 µM	3 d	0.646 ± 0.116	0.798 ± 0.040
11	Blank	0 µM	3 d	1.000 ± 0.282	1.000 ± 0.029
	**OICR-4929**	10 µM	3 d	0.627 ± 0.126	0.692 ± 0.035
	**7k**	10 µM	3 d	1.031 ± 0.214	0.944 ± 0.033
12	Blank	0 µM	3 d	1.000 ± 0.110	1.000 ± 0.029
	**OICR-4929**	10 µM	3 d	0.571 ± 0.059	0.681 ± 0.021
	**7k**	10 µM	3 d	0.548 ± 0.132	0.650 ± 0.065

[a]: Data were also used for concentration dependency experiments (Supplemental Table 7.)

Supplemental Table 7. Raw value of fold change observed from three-day with various concentration of treatment for each individual bioreplicate.

exp label	Condition	conc.	time	HOTTIP	FOXD3-AS1	TGF-β1
				Mean ± SD	Mean ± SD	Mean ± SD
7	Blank	0 µM	3 d	1.000 ± 0.124	1.000 ± 0.106	1.000 ± 0.067
	**OICR-4929**	1 µM	3 d	0.895 ± 0.148	0.873 ± 0.112	0.972 ± 0.070
		5 µM	3 d	0.572 ± 0.081	0.728 ± 0.066	0.873 ± 0.050
		10 µM	3 d	0.680 ± 0.110	0.537 ± 0.055	0.759 ± 0.073
	**7k**	1 µM	3 d	0.923 ± 0.164	0.693 ± 0.147	0.972 ± 0.048
		5 µM	3 d	0.643 ± 0.184	0.753 ± 0.078	0.916 ± 0.056
		10 µM	3 d	0.464 ± 0.082	0.756 ± 0.076	0.972 ± 0.055
8	Blank	0 µM	3 d	1.000 ± 0.023	1.000 ± 0.090	1.000 ± 0.033
	**OICR-4929**	1 µM	3 d	0.846 ± 0.047	0.841 ± 0.071	0.923 ± 0.078
		5 µM	3 d	0.454 ± 0.055	0.636 ± 0.056	0.859 ± 0.050
		10 µM	3 d	0.463 ± 0.056	0.693 ± 0.059	0.982 ± 0.074
	**7k**	1 µM	3 d	0.821 ± 0.069	0.984 ± 0.128	1.020 ± 0.062
		5 µM	3 d	0.877 ± 0.062	1.178 ± 0.131	1.009 ± 0.079
		10 µM	3 d	0.570 ± 0.035	1.075 ± 0.124	0.987 ± 0.059
9	Blank	0 µM	3 d	1.000 ± 0.160	1.000 ± 0.095	1.000 ± 0.139
	**OICR-4929**	1 µM	3 d	0.734 ± 0.094	0.900 ± 0.071	1.029 ± 0.115
		5 µM	3 d	0.578 ± 0.094	0.535 ± 0.043	0.901 ± 0.098
		10 µM	3 d	0.528 ± 0.080	0.615 ± 0.065	0.944 ± 0.112
	**7k**	1 µM	3 d	1.189 ± 0.152	0.971 ± 0.104	1.060 ± 0.127
		5 µM	3 d	0.915 ± 0.153	1.020 ± 0.085	1.003 ± 0.120
		10 µM	3 d	0.733 ± 0.095	0.847 ± 0.077	0.942 ± 0.101

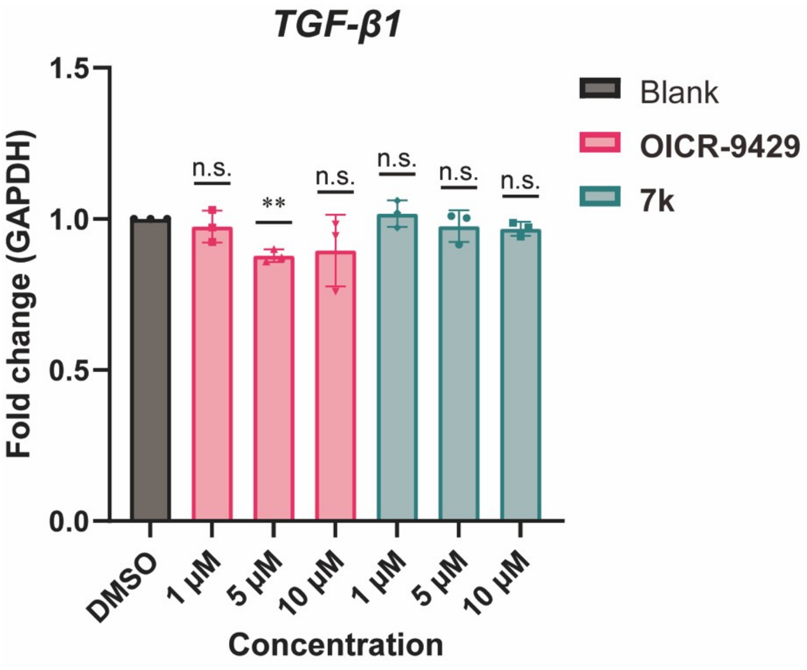

Supplemental Figure 7. Fold change of *TGF-β1* after compound treatment.

Reference

(1) Thieriet, N.; Alsina, J.; Giralt, E.; Guibé, F.; Albericio, F. Use of Alloc-Amino Acids in Solid-Phase Peptide Synthesis. Tandem Deprotection-Coupling Reactions Using Neutral Conditions. *Tetrahedron Letters* **1997**, *38* (41), 7275–7278. https://doi.org/10.1016/S0040-4039(97)01690-0.

(2) Chang, J.-Y.; Neugebauer, C.; Schmeing, S.; Amrahova, G.; Hart, P. ’t. Macrocyclic Peptides as Inhibitors of WDR5 - lncRNA Interactions. *Chem. Commun.* **2023**. https://doi.org/10.1039/D3CC03221C.

(3) Chatterjee, J.; Laufer, B.; Kessler, H. Synthesis of N-Methylated Cyclic Peptides. *Nat Protoc* **2012**, *7* (3), 432–444. https://doi.org/10.1038/nprot.2011.450.

(4) Fukuyama, T.; Cheung, M.; Kan, T. N-Carboalkoxy-2-Nitrobenzenesulfonamides: A Practical Preparation of N-Boc-, N-Alloc-, and N-Cbz-Protected Primary Amines. *Synlett* **1999**, *1999* (8), 1301–1303. https://doi.org/10.1055/s-1999-2827.

(5) Nikolovska-Coleska, Z.; Wang, R.; Fang, X.; Pan, H.; Tomita, Y.; Li, P.; Roller, P. P.; Krajewski, K.; Saito, N. G.; Stuckey, J. A.; Wang, S. Development and Optimization of a Binding Assay for the XIAP BIR3 Domain Using Fluorescence Polarization. *Analytical Biochemistry* **2004**, *332* (2), 261–273. https://doi.org/10.1016/j.ab.2004.05.055.

(6) Fuentes-Iglesias, A.; Garcia-Outeiral, V.; Pardavila, J. A.; Wang, J.; Fidalgo, M.; Guallar, D. An Optimized Immunoprecipitation Protocol for Assessing Protein-RNA Interactions In Vitro. *STAR Protocols* **2020**, *1* (2), 100093. https://doi.org/10.1016/j.xpro.2020.100093.

(7) Livak, K. J.; Schmittgen, T. D. Analysis of Relative Gene Expression Data Using Real-Time Quantitative PCR and the 2−ΔΔCT Method. *Methods* **2001**, *25* (4), 402–408. https://doi.org/10.1006/meth.2001.1262.

(8) Chou, P.-H.; Wang, S.-T.; Ma, H.-L.; Liu, C.-L.; Chang, M.-C.; Lee, O. K.-S. Development of a Two-Step Protocol for Culture Expansion of Human Annulus Fibrosus Cells with TGF-Β1 and FGF-2. *Stem Cell Research & Therapy* **2016**, *7* (1), 89. https://doi.org/10.1186/s13287-016-0332-1.

(9) Iempridee, T.; Wiwithaphon, S.; Piboonprai, K.; Pratedrat, P.; Khumkhrong, P.; Japrung, D.; Temisak, S.; Laiwejpithaya, S.; Chaopotong, P.; Dharakul, T. Identification of Reference Genes for Circulating Long Noncoding RNA Analysis in Serum of Cervical Cancer Patients. *FEBS Open Bio* **2018**, *8* (11), 1844–1854. https://doi.org/10.1002/2211-5463.12523.

(10) Li, Y.; Liu, Y.; Chen, G.; Liu, H.; Wu, Y.; Liu, J.; Zhang, Z. HOTTIP Is Upregulated in Esophageal Cancer and Triggers the Drug Resistance.

(11) Subhash, S.; Mishra, K.; Akhade, V. S.; Kanduri, M.; Mondal, T.; Kanduri, C. H3K4me2 and WDR5 Enriched Chromatin Interacting Long Non-Coding RNAs Maintain Transcriptionally

Competent Chromatin at Divergent Transcriptional Units. *Nucleic Acids Research* **2018**, *46* (18), 9384–9400. https://doi.org/10.1093/nar/gky635.

(12) Scavello, F.; Zeni, F.; Milano, G.; Macrì, F.; Castiglione, S.; Zuccolo, E.; Scopece, A.; Pezone, G.; Tedesco, C. C.; Nigro, P.; Degani, G.; Gambini, E.; Veglia, F.; Popolo, L.; Pompilio, G.; Colombo, G. I.; Bianchi, M. E.; Raucci, A. Soluble Receptor for Advanced Glycation End-Products Regulates Age-Associated Cardiac Fibrosis. *International Journal of Biological Sciences* **2021**, *17* (10), 2399–2416. https://doi.org/10.7150/ijbs.56379.
